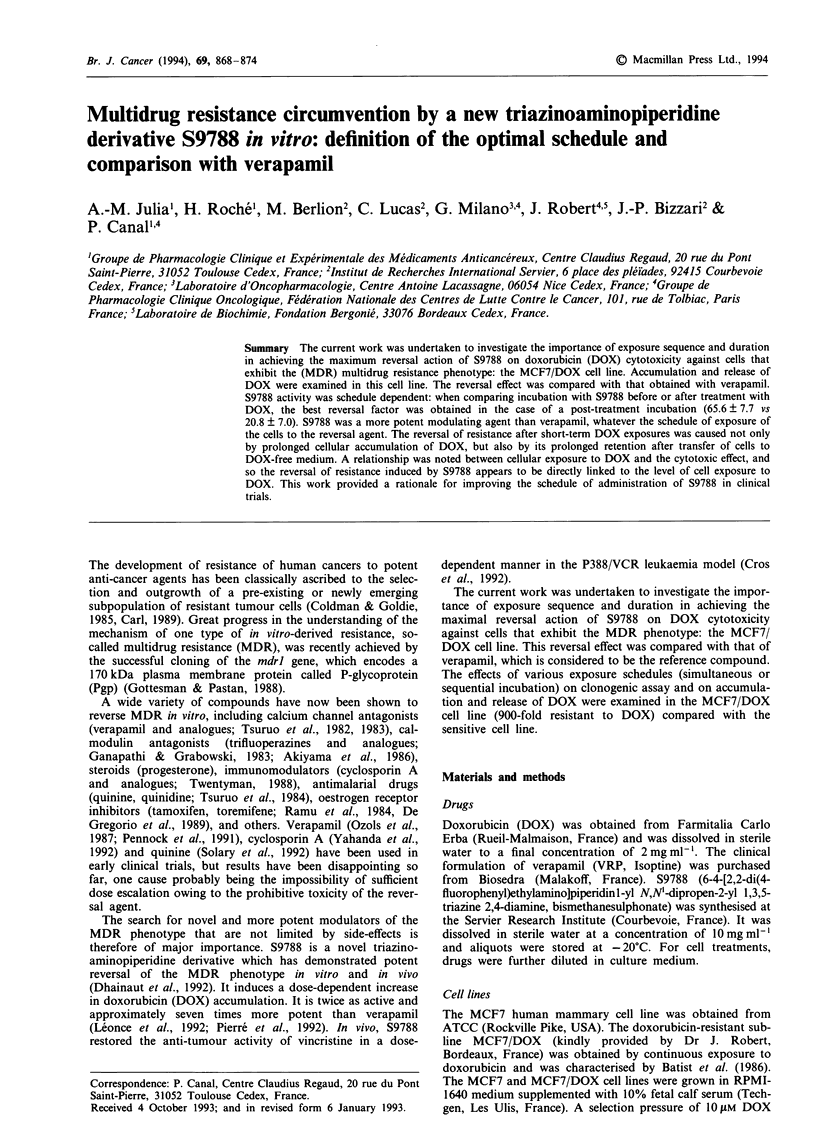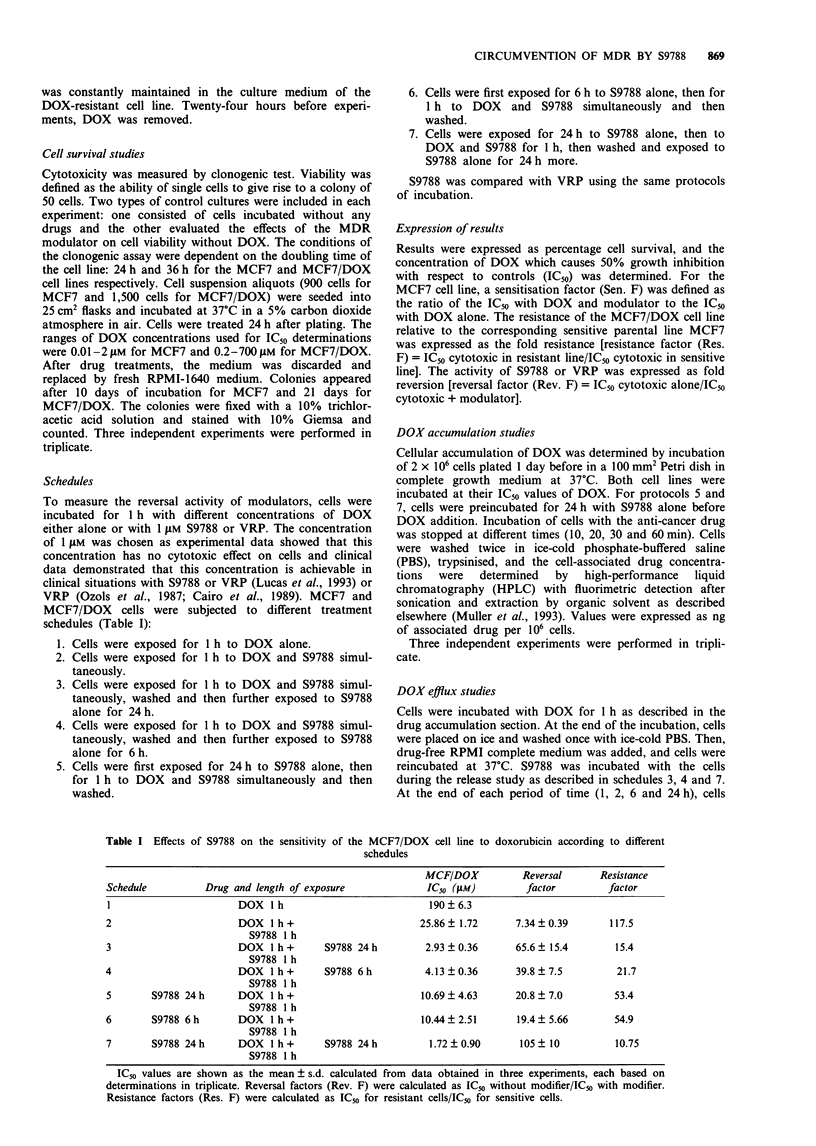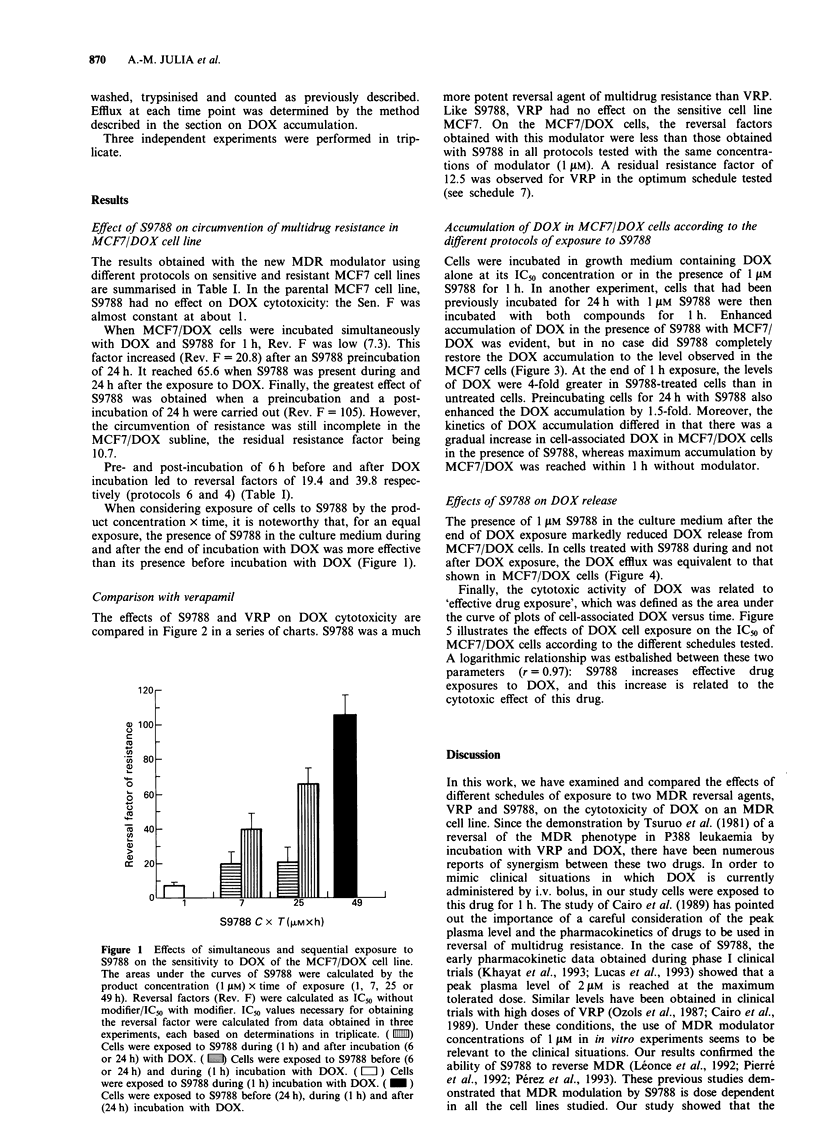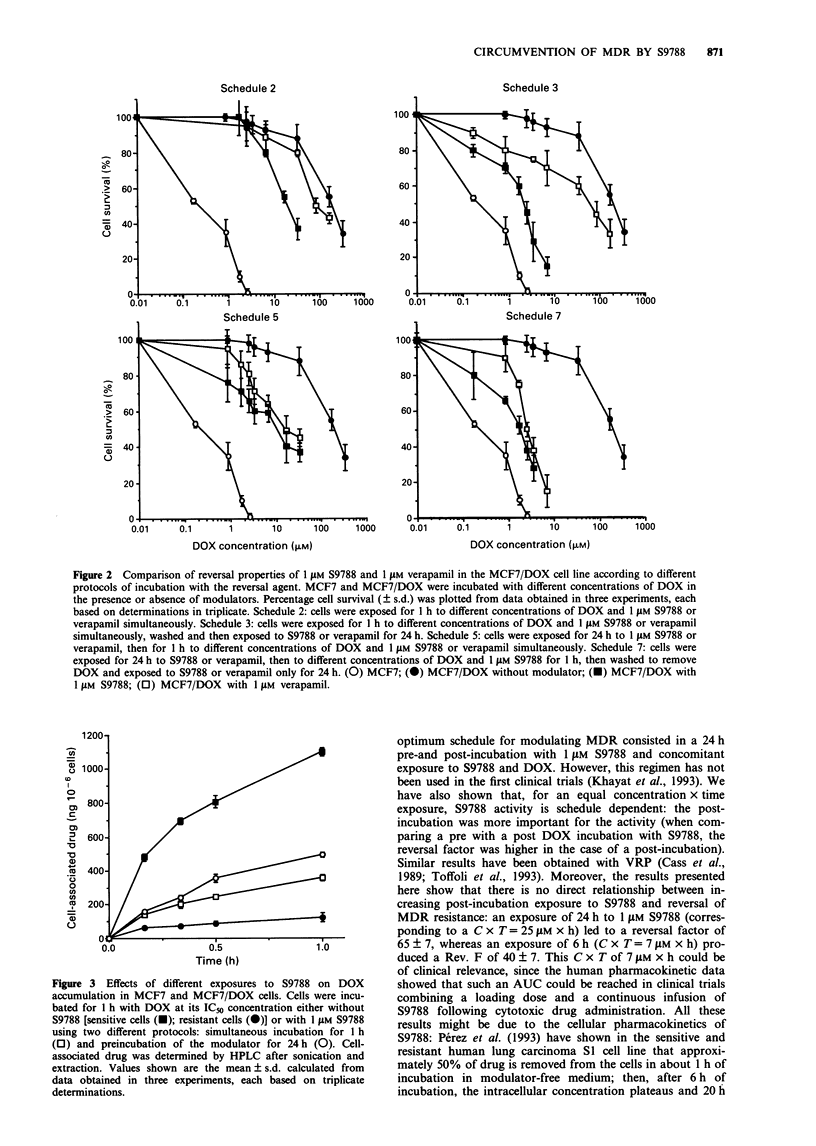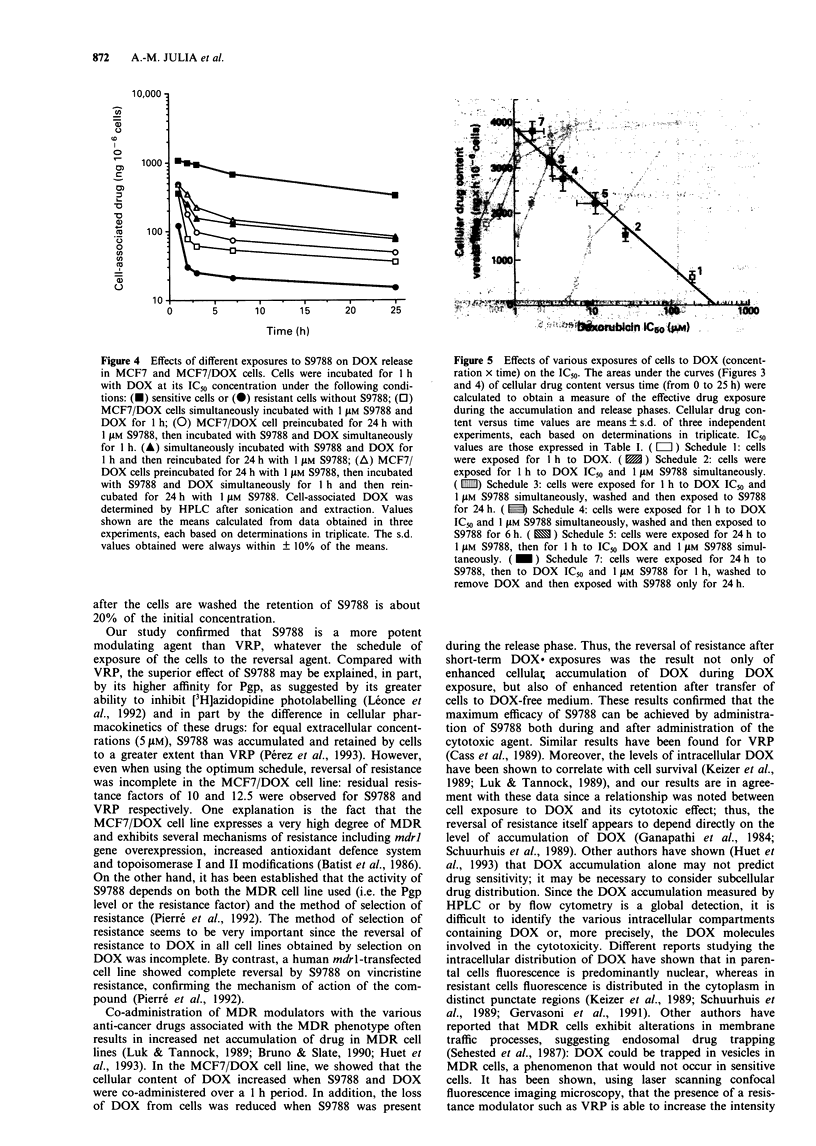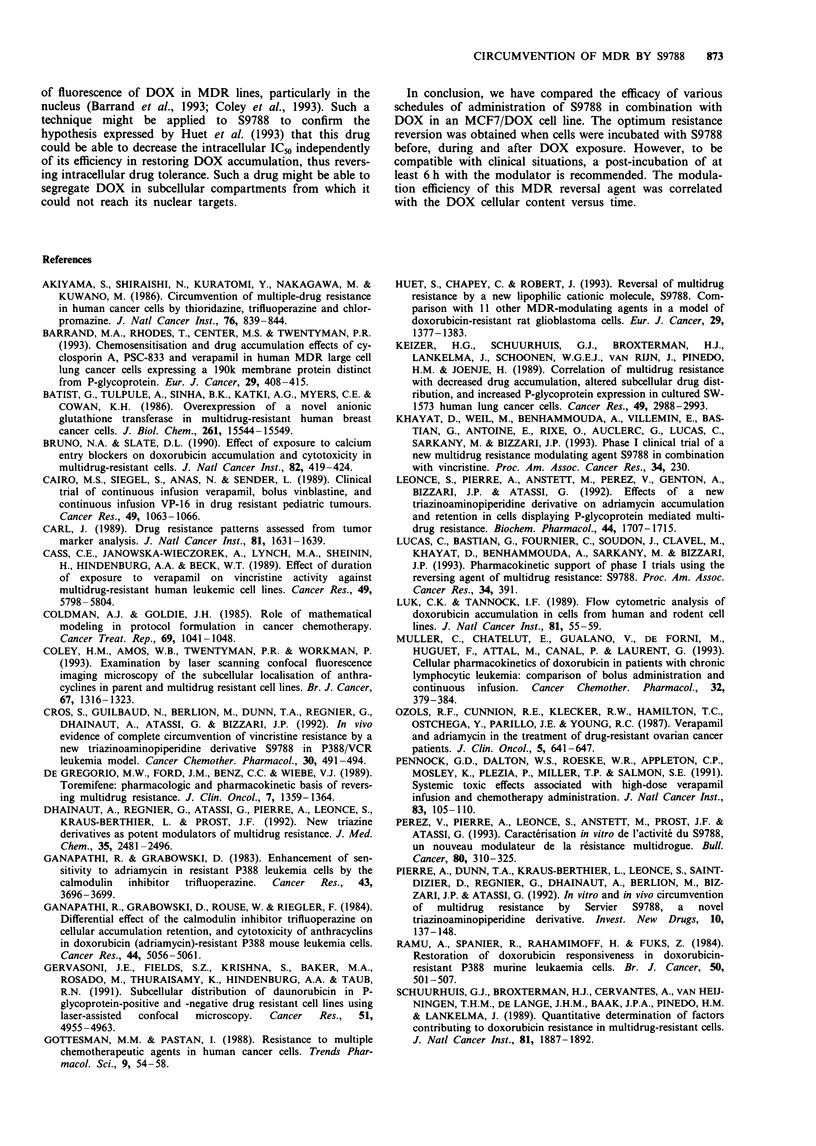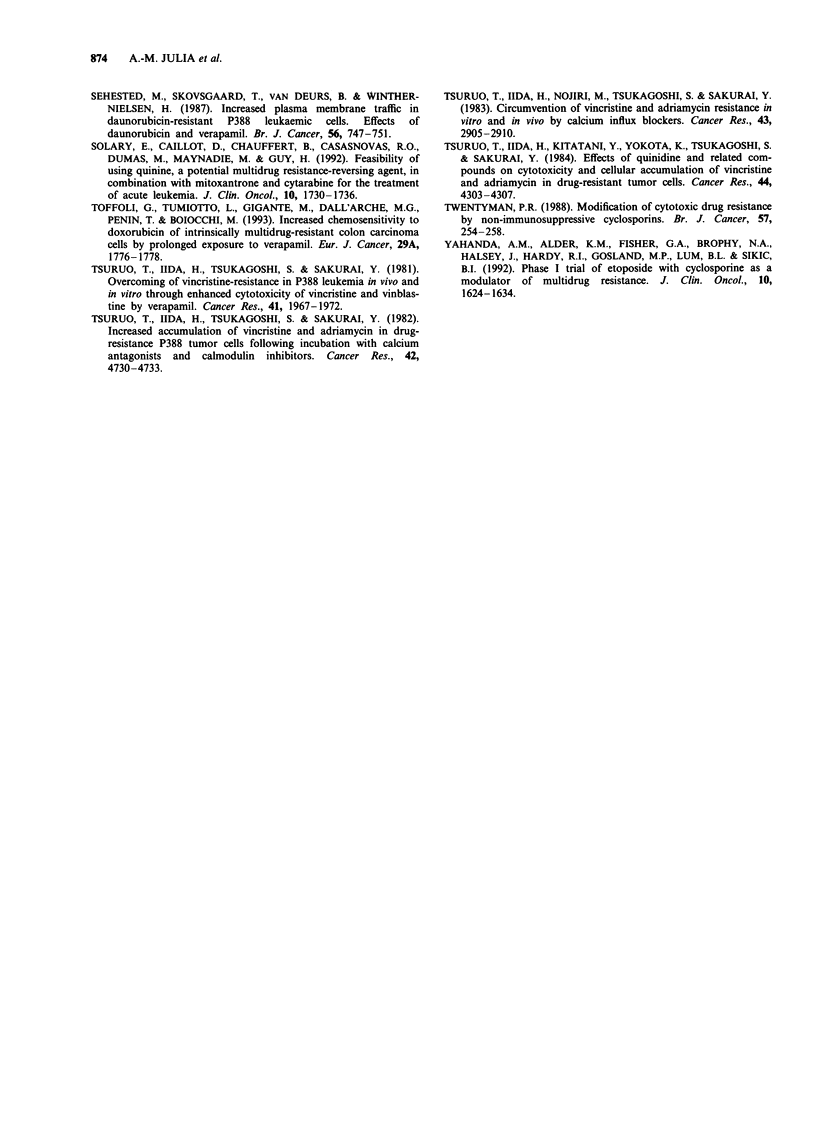# Multidrug resistance circumvention by a new triazinoaminopiperidine derivative S9788 in vitro: definition of the optimal schedule and comparison with verapamil.

**DOI:** 10.1038/bjc.1994.168

**Published:** 1994-05

**Authors:** A. M. Julia, H. Roché, M. Berlion, C. Lucas, G. Milano, J. Robert, J. P. Bizzari, P. Canal

**Affiliations:** Centre Claudius Regaud, Toulouse, France.

## Abstract

The current work was undertaken to investigate the importance of exposure sequence and duration in achieving the maximum reversal action of S9788 on doxorubicin (DOX) cytotoxicity against cells that exhibit the (MDR) multidrug resistance phenotype: the MCF7/DOX cell line. Accumulation and release of DOX were examined in this cell line. The reversal effect was compared with that obtained with verapamil. S9788 activity was schedule dependent: when comparing incubation with S9788 before or after treatment with DOX, the best reversal factor was obtained in the case of a post-treatment incubation (65.6 +/- 7.7 vs 20.8 +/- 7.0). S9788 was a more potent modulating agent than verapamil, whatever the schedule of exposure of the cells to the reversal agent. The reversal of resistance after short-term DOX exposures was caused not only by prolonged cellular accumulation of DOX, but also by its prolonged retention after transfer of cells to DOX-free medium. A relationship was noted between cellular exposure to DOX and the cytotoxic effect, and so the reversal of resistance induced by S9788 appears to be directly linked to the level of cell exposure to DOX. This work provided a rationale for improving the schedule of administration of S9788 in clinical trials.


Br. J. Cancer (1994), 69, 868-874 © Macmillan Press Ltd., 1994

### Multidrug resistance circumvention by a new triazinoaminopiperidine derivative S9788 *in vitro*: definition of the optimal schedule and comparison with verapamil

A.-M. Julia[1], H. Roché[1], M. Berlion[2], C. Lucas[2], G. Milano[3,4], J. Robert[4,5], J.-P. Bizzari[2] & P. Canal[1,4]

[1]*Groupe de Pharmacologie Clinique et Expérimentale des Médicaments Anticancéreux, Centre Claudius Regaud, 20 rue du Pont Saint-Pierre, 31052 Toulouse Cedex, France;* [2]*Institut de Recherches International Servier, 6 place des pléïades, 92415 Courbevoie Cedex, France;* [3]*Laboratoire d'Oncopharmacologie, Centre Antoine Lacassagne, 06054 Nice Cedex, France;* [4]*Groupe de Pharmacologie Clinique Oncologique, Fédération Nationale des Centres de Lutte Contre le Cancer, 101, rue de Tolbiac, Paris France;* [5]*Laboratoire de Biochimie, Fondation Bergonié, 33076 Bordeaux Cedex, France.*

**Summary** The current work was undertaken to investigate the importance of exposure sequence and duration in achieving the maximum reversal action of S9788 on doxorubicin (DOX) cytotoxicity against cells that exhibit the (MDR) multidrug resistance phenotype: the MCF7/DOX cell line. Accumulation and release of DOX were examined in this cell line. The reversal effect was compared with that obtained with verapamil. S9788 activity was schedule dependent: when comparing incubation with S9788 before or after treatment with DOX, the best reversal factor was obtained in the case of a post-treatment incubation (65.6 ± 7.7 *vs* 20.8 ± 7.0). S9788 was a more potent modulating agent than verapamil, whatever the schedule of exposure of the cells to the reversal agent. The reversal of resistance after short-term DOX exposures was caused not only by prolonged cellular accumulation of DOX, but also by its prolonged retention after transfer of cells to DOX-free medium. A relationship was noted between cellular exposure to DOX and the cytotoxic effect, and so the reversal of resistance induced by S9788 appears to be directly linked to the level of cell exposure to DOX. This work provided a rationale for improving the schedule of administration of S9788 in clinical trials.

The development of resistance of human cancers to potent anti-cancer agents has been classically ascribed to the selection and outgrowth of a pre-existing or newly emerging subpopulation of resistant tumour cells (Coldman & Goldie, 1985, Carl, 1989). Great progress in the understanding of the mechanism of one type of *in vitro*-derived resistance, so-called multidrug resistance (MDR), was recently achieved by the successful cloning of the *mdr1* gene, which encodes a 170 kDa plasma membrane protein called P-glycoprotein (Pgp) (Gottesman & Pastan, 1988).

A wide variety of compounds have now been shown to reverse MDR *in vitro*, including calcium channel antagonists (verapamil and analogues; Tsuruo *et al.*, 1982, 1983), calmodulin antagonists (trifluoperazines and analogues; Ganapathi & Grabowski, 1983; Akiyama *et al.*, 1986), steroids (progesterone), immunomodulators (cyclosporin A and analogues; Twentyman, 1988), antimalarial drugs (quinine, quinidine; Tsuruo *et al.*, 1984), oestrogen receptor inhibitors (tamoxifen, toremifene; Ramu *et al.*, 1984, De Gregorio *et al.*, 1989), and others. Verapamil (Ozols *et al.*, 1987; Pennock *et al.*, 1991), cyclosporin A (Yahanda *et al.*, 1992) and quinine (Solary *et al.*, 1992) have been used in early clinical trials, but results have been disappointing so far, one cause probably being the impossibility of sufficient dose escalation owing to the prohibitive toxicity of the reversal agent.

The search for novel and more potent modulators of the MDR phenotype that are not limited by side-effects is therefore of major importance. S9788 is a novel triazinoaminopiperidine derivative which has demonstrated potent reversal of the MDR phenotype *in vitro* and *in vivo* (Dhainaut *et al.*, 1992). It induces a dose-dependent increase in doxorubicin (DOX) accumulation. It is twice as active and approximately seven times more potent than verapamil (Léonce *et al.*, 1992; Pierré *et al.*, 1992). *In vivo*, S9788 restored the anti-tumour activity of vincristine in a dose-dependent manner in the P388/VCR leukaemia model (Cros *et al.*, 1992).

The current work was undertaken to investigate the importance of exposure sequence and duration in achieving the maximal reversal action of S9788 on DOX cytotoxicity against cells that exhibit the MDR phenotype: the MCF7/DOX cell line. This reversal effect was compared with that of verapamil, which is considered to be the reference compound. The effects of various exposure schedules (simultaneous or sequential incubation) on clonogenic assay and on accumulation and release of DOX were examined in the MCF7/DOX cell line (900-fold resistant to DOX) compared with the sensitive cell line.

### Materials and methods

### *Drugs*

Doxorubicin (DOX) was obtained from Farmitalia Carlo Erba (Rueil-Malmaison, France) and was dissolved in sterile water to a final concentration of 2 mg ml$^{-1}$. The clinical formulation of verapamil (VRP, Isoptine) was purchased from Biosedra (Malakoff, France). S9788 (6-4-[2,2-di(4-fluorophenyl)ethylamino]piperidin1-yl *N*,*N*$^1$-dipropen-2-yl 1,3,5-triazine 2,4-diamine, bismethanesulphonate) was synthesised at the Servier Research Institute (Courbevoie, France). It was dissolved in sterile water at a concentration of 10 mg ml$^{-1}$ and aliquots were stored at −20°C. For cell treatments, drugs were further diluted in culture medium.

### *Cell lines*

The MCF7 human mammary cell line was obtained from ATCC (Rockville Pike, USA). The doxorubicin-resistant subline MCF7/DOX (kindly provided by Dr J. Robert, Bordeaux, France) was obtained by continuous exposure to doxorubicin and was characterised by Batist *et al.* (1986). The MCF7 and MCF7/DOX cell lines were grown in RPMI-1640 medium supplemented with 10% fetal calf serum (Techgen, Les Ulis, France). A selection pressure of 10 μM DOX

Correspondence: P. Canal, Centre Claudius Regaud, 20 rue du Pont Saint-Pierre, 31052 Toulouse Cedex, France.
Received 4 October 1993; and in revised form 6 January 1993.

was constantly maintained in the culture medium of the DOX-resistant cell line. Twenty-four hours before experiments, DOX was removed.

*Cell survival studies*

Cytotoxicity was measured by clonogenic test. Viability was defined as the ability of single cells to give rise to a colony of 50 cells. Two types of control cultures were included in each experiment: one consisted of cells incubated without any drugs and the other evaluated the effects of the MDR modulator on cell viability without DOX. The conditions of the clonogenic assay were dependent on the doubling time of the cell line: 24 h and 36 h for the MCF7 and MCF7/DOX cell lines respectively. Cell suspension aliquots (900 cells for MCF7 and 1,500 cells for MCF7/DOX) were seeded into 25 $cm^2$ flasks and incubated at 37°C in a 5% carbon dioxide atmosphere in air. Cells were treated 24 h after plating. The ranges of DOX concentrations used for $IC_{50}$ determinations were 0.01–2 μM for MCF7 and 0.2–700 μM for MCF7/DOX. After drug treatments, the medium was discarded and replaced by fresh RPMI-1640 medium. Colonies appeared after 10 days of incubation for MCF7 and 21 days for MCF7/DOX. The colonies were fixed with a 10% trichloracetic acid solution and stained with 10% Giemsa and counted. Three independent experiments were performed in triplicate.

*Schedules*

To measure the reversal activity of modulators, cells were incubated for 1 h with different concentrations of DOX either alone or with 1 μM S9788 or VRP. The concentration of 1 μM was chosen as experimental data showed that this concentration has no cytotoxic effect on cells and clinical data demonstrated that this concentration is achievable in clinical situations with S9788 or VRP (Lucas *et al.*, 1993) or VRP (Ozols *et al.*, 1987; Cairo *et al.*, 1989). MCF7 and MCF7/DOX cells were subjected to different treatment schedules (Table I):

1. Cells were exposed for 1 h to DOX alone.
2. Cells were exposed for 1 h to DOX and S9788 simultaneously.
3. Cells were exposed for 1 h to DOX and S9788 simultaneously, washed and then further exposed to S9788 alone for 24 h.
4. Cells were exposed for 1 h to DOX and S9788 simultaneously, washed and then further exposed to S9788 alone for 6 h.
5. Cells were first exposed for 24 h to S9788 alone, then for 1 h to DOX and S9788 simultaneously and then washed.
6. Cells were first exposed for 6 h to S9788 alone, then for 1 h to DOX and S9788 simultaneously and then washed.
7. Cells were exposed for 24 h to S9788 alone, then to DOX and S9788 for 1 h, then washed and exposed to S9788 alone for 24 h more.

S9788 was compared with VRP using the same protocols of incubation.

*Expression of results*

Results were expressed as percentage cell survival, and the concentration of DOX which causes 50% growth inhibition with respect to controls ($IC_{50}$) was determined. For the MCF7 cell line, a sensitisation factor (Sen. F) was defined as the ratio of the $IC_{50}$ with DOX and modulator to the $IC_{50}$ with DOX alone. The resistance of the MCF7/DOX cell line relative to the corresponding sensitive parental line MCF7 was expressed as the fold resistance [resistance factor (Res. F) = $IC_{50}$ cytotoxic in resistant line/$IC_{50}$ cytotoxic in sensitive line]. The activity of S9788 or VRP was expressed as fold reversion [reversal factor (Rev. F) = $IC_{50}$ cytotoxic alone/$IC_{50}$ cytotoxic + modulator].

*DOX accumulation studies*

Cellular accumulation of DOX was determined by incubation of $2 \times 10^6$ cells plated 1 day before in a 100 $mm^2$ Petri dish in complete growth medium at 37°C. Both cell lines were incubated at their $IC_{50}$ values of DOX. For protocols 5 and 7, cells were preincubated for 24 h with S9788 alone before DOX addition. Incubation of cells with the anti-cancer drug was stopped at different times (10, 20, 30 and 60 min). Cells were washed twice in ice-cold phosphate-buffered saline (PBS), trypsinised, and the cell-associated drug concentrations were determined by high-performance liquid chromatography (HPLC) with fluorimetric detection after sonication and extraction by organic solvent as described elsewhere (Muller *et al.*, 1993). Values were expressed as ng of associated drug per $10^6$ cells.

Three independent experiments were performed in triplicate.

*DOX efflux studies*

Cells were incubated with DOX for 1 h as described in the drug accumulation section. At the end of the incubation, cells were placed on ice and washed once with ice-cold PBS. Then, drug-free RPMI complete medium was added, and cells were reincubated at 37°C. S9788 was incubated with the cells during the release study as described in schedules 3, 4 and 7. At the end of each period of time (1, 2, 6 and 24 h), cells

**Table I** Effects of S9788 on the sensitivity of the MCF7/DOX cell line to doxorubicin according to different schedules

| *Schedule* | *Drug and length of exposure* | | | *MCF/DOX $IC_{50}$ (μM)* | *Reversal factor* | *Resistance factor* |
|---|---|---|---|---|---|---|
| 1 | | DOX 1 h | | 190 ± 6.3 | | |
| 2 | | DOX 1 h + S9788 1 h | | 25.86 ± 1.72 | 7.34 ± 0.39 | 117.5 |
| 3 | | DOX 1 h + S9788 1 h | S9788 24 h | 2.93 ± 0.36 | 65.6 ± 15.4 | 15.4 |
| 4 | | DOX 1 h + S9788 1 h | S9788 6 h | 4.13 ± 0.36 | 39.8 ± 7.5 | 21.7 |
| 5 | S9788 24 h | DOX 1 h + S9788 1 h | | 10.69 ± 4.63 | 20.8 ± 7.0 | 53.4 |
| 6 | S9788 6 h | DOX 1 h + S9788 1 h | | 10.44 ± 2.51 | 19.4 ± 5.66 | 54.9 |
| 7 | S9788 24 h | DOX 1 h + S9788 1 h | S9788 24 h | 1.72 ± 0.90 | 105 ± 10 | 10.75 |

$IC_{50}$ values are shown as the mean ± s.d. calculated from data obtained in three experiments, each based on determinations in triplicate. Reversal factors (Rev. F) were calculated as $IC_{50}$ without modifier/$IC_{50}$ with modifier. Resistance factors (Res. F) were calculated as $IC_{50}$ for resistant cells/$IC_{50}$ for sensitive cells.

washed, trypsinised and counted as previously described. Efflux at each time point was determined by the method described in the section on DOX accumulation.

Three independent experiments were performed in triplicate.

### Results

### *Effect of S9788 on circumvention of multidrug resistance in MCF7/DOX cell line*

The results obtained with the new MDR modulator using different protocols on sensitive and resistant MCF7 cell lines are summarised in Table I. In the parental MCF7 cell line, S9788 had no effect on DOX cytotoxicity: the Sen. F was almost constant at about 1.

When MCF7/DOX cells were incubated simultaneously with DOX and S9788 for 1 h, Rev. F was low (7.3). This factor increased (Rev. F = 20.8) after an S9788 preincubation of 24 h. It reached 65.6 when S9788 was present during and 24 h after the exposure to DOX. Finally, the greatest effect of S9788 was obtained when a preincubation and a post-incubation of 24 h were carried out (Rev. F = 105). However, the circumvention of resistance was still incomplete in the MCF7/DOX subline, the residual resistance factor being 10.7.

Pre- and post-incubation of 6 h before and after DOX incubation led to reversal factors of 19.4 and 39.8 respectively (protocols 6 and 4) (Table I).

When considering exposure of cells to S9788 by the product concentration × time, it is noteworthy that, for an equal exposure, the presence of S9788 in the culture medium during and after the end of incubation with DOX was more effective than its presence before incubation with DOX (Figure 1).

### *Comparison with verapamil*

The effects of S9788 and VRP on DOX cytotoxicity are compared in Figure 2 in a series of charts. S9788 was a much more potent reversal agent of multidrug resistance than VRP. Like S9788, VRP had no effect on the sensitive cell line MCF7. On the MCF7/DOX cells, the reversal factors obtained with this modulator were less than those obtained with S9788 in all protocols tested with the same concentrations of modulator (1 μM). A residual resistance factor of 12.5 was observed for VRP in the optimum schedule tested (see schedule 7).

**Figure 1** Effects of simultaneous and sequential exposure to S9788 on the sensitivity to DOX of the MCF7/DOX cell line. The areas under the curves of S9788 were calculated by the product concentration (1 μM) × time of exposure (1, 7, 25 or 49 h). Reversal factors (Rev. F) were calculated as $IC_{50}$ without modifier/$IC_{50}$ with modifier. $IC_{50}$ values necessary for obtaining the reversal factor were calculated from data obtained in three experiments, each based on determinations in triplicate. (▥) Cells were exposed to S9788 during (1 h) and after incubation (6 or 24 h) with DOX. (▤) Cells were exposed to S9788 before (6 or 24 h) and during (1 h) incubation with DOX. (□) Cells were exposed to S9788 during (1 h) incubation with DOX. (■) Cells were exposed to S9788 before (24 h), during (1 h) and after (24 h) incubation with DOX.

### *Accumulation of DOX in MCF7/DOX cells according to the different protocols of exposure to S9788*

Cells were incubated in growth medium containing DOX alone at its $IC_{50}$ concentration or in the presence of 1 μM S9788 for 1 h. In another experiment, cells that had been previously incubated for 24 h with 1 μM S9788 were then incubated with both compounds for 1 h. Enhanced accumulation of DOX in the presence of S9788 with MCF7/DOX was evident, but in no case did S9788 completely restore the DOX accumulation to the level observed in the MCF7 cells (Figure 3). At the end of 1 h exposure, the levels of DOX were 4-fold greater in S9788-treated cells than in untreated cells. Preincubating cells for 24 h with S9788 also enhanced the DOX accumulation by 1.5-fold. Moreover, the kinetics of DOX accumulation differed in that there was a gradual increase in cell-associated DOX in MCF7/DOX cells in the presence of S9788, whereas maximum accumulation by MCF7/DOX was reached within 1 h without modulator.

### *Effects of S9788 on DOX release*

The presence of 1 μM S9788 in the culture medium after the end of DOX exposure markedly reduced DOX release from MCF7/DOX cells. In cells treated with S9788 during and not after DOX exposure, the DOX efflux was equivalent to that shown in MCF7/DOX cells (Figure 4).

Finally, the cytotoxic activity of DOX was related to 'effective drug exposure', which was defined as the area under the curve of plots of cell-associated DOX versus time. Figure 5 illustrates the effects of DOX cell exposure on the $IC_{50}$ of MCF7/DOX cells according to the different schedules tested. A logarithmic relationship was estbalished between these two parameters ($r = 0.97$): S9788 increases effective drug exposures to DOX, and this increase is related to the cytotoxic effect of this drug.

### Discussion

In this work, we have examined and compared the effects of different schedules of exposure to two MDR reversal agents, VRP and S9788, on the cytotoxicity of DOX on an MDR cell line. Since the demonstration by Tsuruo *et al.* (1981) of a reversal of the MDR phenotype in P388 leukaemia by incubation with VRP and DOX, there have been numerous reports of synergism between these two drugs. In order to mimic clinical situations in which DOX is currently administered by i.v. bolus, in our study cells were exposed to this drug for 1 h. The study of Cairo *et al.* (1989) has pointed out the importance of a careful consideration of the peak plasma level and the pharmacokinetics of drugs to be used in reversal of multidrug resistance. In the case of S9788, the early pharmacokinetic data obtained during phase I clinical trials (Khayat *et al.*, 1993; Lucas *et al.*, 1993) showed that a peak plasma level of 2 μM is reached at the maximum tolerated dose. Similar levels have been obtained in clinical trials with high doses of VRP (Ozols *et al.*, 1987; Cairo *et al.*, 1989). Under these conditions, the use of MDR modulator concentrations of 1 μM in *in vitro* experiments seems to be relevant to the clinical situations. Our results confirmed the ability of S9788 to reverse MDR (Léonce *et al.*, 1992; Pierré *et al.*, 1992; Pérez *et al.*, 1993). These previous studies demonstrated that MDR modulation by S9788 is dose dependent in all the cell lines studied. Our study showed that the

**Figure 2** Comparison of reversal properties of 1 μM S9788 and 1 μM verapamil in the MCF7/DOX cell line according to different protocols of incubation with the reversal agent. MCF7 and MCF7/DOX were incubated with different concentrations of DOX in the presence or absence of modulators. Percentage cell survival (± s.d.) was plotted from data obtained in three experiments, each based on determinations in triplicate. Schedule 2: cells were exposed for 1 h to different concentrations of DOX and 1 μM S9788 or verapamil simultaneously. Schedule 3: cells were exposed for 1 h to different concentrations of DOX and 1 μM S9788 or verapamil simultaneously, washed and then exposed to S9788 or verapamil for 24 h. Schedule 5: cells were exposed for 24 h to 1 μM S9788 or verapamil, then for 1 h to different concentrations of DOX and 1 μM S9788 or verapamil simultaneously. Schedule 7: cells were exposed for 24 h to S9788 or verapamil, then to different concentrations of DOX and 1 μM S9788 for 1 h, then washed to remove DOX and exposed to S9788 or verapamil only for 24 h. (○) MCF7; (●) MCF7/DOX without modulator; (■) MCF7/DOX with 1 μM S9788; (□) MCF7/DOX with 1 μM verapamil.

**Figure 3** Effects of different exposures to S9788 on DOX accumulation in MCF7 and MCF7/DOX cells. Cells were incubated for 1 h with DOX at its $IC_{50}$ concentration either without S9788 [sensitive cells (■); resistant cells (●)] or with 1 μM S9788 using two different protocols: simultaneous incubation for 1 h (□) and preincubation of the modulator for 24 h (○). Cell-associated drug was determined by HPLC after sonication and extraction. Values shown are the mean ± s.d. calculated from data obtained in three experiments, each based on triplicate determinations.

optimum schedule for modulating MDR consisted in a 24 h pre-and post-incubation with 1 μM S9788 and concomitant exposure to S9788 and DOX. However, this regimen has not been used in the first clinical trials (Khayat *et al.*, 1993). We have also shown that, for an equal concentration × time exposure, S9788 activity is schedule dependent: the post-incubation was more important for the activity (when comparing a pre with a post DOX incubation with S9788, the reversal factor was higher in the case of a post-incubation). Similar results have been obtained with VRP (Cass *et al.*, 1989; Toffoli *et al.*, 1993). Moreover, the results presented here show that there is no direct relationship between increasing post-incubation exposure to S9788 and reversal of MDR resistance: an exposure of 24 h to 1 μM S9788 (corresponding to a $C \times T = 25$ μM × h) led to a reversal factor of 65 ± 7, whereas an exposure of 6 h ($C \times T = 7$ μM × h) produced a Rev. F of 40 ± 7. This $C \times T$ of 7 μM × h could be of clinical relevance, since the human pharmacokinetic data showed that such an AUC could be reached in clinical trials combining a loading dose and a continuous infusion of S9788 following cytotoxic drug administration. All these results might be due to the cellular pharmacokinetics of S9788: Pérez *et al.* (1993) have shown in the sensitive and resistant human lung carcinoma S1 cell line that approximately 50% of drug is removed from the cells in about 1 h of incubation in modulator-free medium; then, after 6 h of incubation, the intracellular concentration plateaus and 20 h

**Figure 4** Effects of different exposures to S9788 on DOX release in MCF7 and MCF7/DOX cells. Cells were incubated for 1 h with DOX at its $IC_{50}$ concentration under the following conditions: (■) sensitive cells or (●) resistant cells without S9788; (□) MCF7/DOX cells simultaneously incubated with 1 μM S9788 and DOX for 1 h; (○) MCF7/DOX cell preincubated for 24 h with 1 μM S9788, then incubated with S9788 and DOX simultaneously for 1 h. (▲) simultaneously incubated with S9788 and DOX for 1 h and then reincubated for 24 h with 1 μM S9788; (△) MCF7/DOX cells preincubated for 24 h with 1 μM S9788, then incubated with S9788 and DOX simultaneously for 1 h and then reincubated for 24 h with 1 μM S9788. Cell-associated DOX was determined by HPLC after sonication and extraction. Values shown are the means calculated from data obtained in three experiments, each based on determinations in triplicate. The s.d. values obtained were always within ± 10% of the means.

**Figure 5** Effects of various exposures of cells to DOX (concentration × time) on the $IC_{50}$. The areas under the curves (Figures 3 and 4) of cellular drug content versus time (from 0 to 25 h) were calculated to obtain a measure of the effective drug exposure during the accumulation and release phases. Cellular drug content versus time values are means ± s.d. of three independent experiments, each based on determinations in triplicate. $IC_{50}$ values are those expressed in Table I. (□) Schedule 1: cells were exposed for 1 h to DOX. (▨) Schedule 2: cells were exposed for 1 h to DOX $IC_{50}$ and 1 μM S9788 simultaneously. (▥) Schedule 3: cells were exposed for 1 h to DOX $IC_{50}$ and 1 μM S9788 simultaneously, washed and then exposed to S9788 for 24 h. (▤) Schedule 4: cells were exposed for 1 h to DOX $IC_{50}$ and 1 μM S9788 simultaneously, washed and then exposed to S9788 for 6 h. (▧) Schedule 5: cells were exposed for 24 h to 1 μM S9788, then for 1 h to $IC_{50}$ DOX and 1 μM S9788 simultaneously. (■) Schedule 7: cells were exposed for 24 h to S9788, then to DOX $IC_{50}$ and 1 μM S9788 for 1 h, washed to remove DOX and then exposed with S9788 only for 24 h.

after the cells are washed the retention of S9788 is about 20% of the initial concentration.

Our study confirmed that S9788 is a more potent modulating agent than VRP, whatever the schedule of exposure of the cells to the reversal agent. Compared with VRP, the superior effect of S9788 may be explained, in part, by its higher affinity for Pgp, as suggested by its greater ability to inhibit [$^3$H]azidopidine photolabelling (Léonce et al., 1992) and in part by the difference in cellular pharmacokinetics of these drugs: for equal extracellular concentrations (5 μM), S9788 was accumulated and retained by cells to a greater extent than VRP (Pérez et al., 1993). However, even when using the optimum schedule, reversal of resistance was incomplete in the MCF7/DOX cell line: residual resistance factors of 10 and 12.5 were observed for S9788 and VRP respectively. One explanation is the fact that the MCF7/DOX cell line expresses a very high degree of MDR and exhibits several mechanisms of resistance including *mdrl* gene overexpression, increased antioxidant defence system and topoisomerase I and II modifications (Batist et al., 1986). On the other hand, it has been established that the activity of S9788 depends on both the MDR cell line used (i.e. the Pgp level or the resistance factor) and the method of selection of resistance (Pierré et al., 1992). The method of selection of resistance seems to be very important since the reversal of resistance to DOX in all cell lines obtained by selection on DOX was incomplete. By contrast, a human *mdr*1-transfected cell line showed complete reversal by S9788 on vincristine resistance, confirming the mechanism of action of the compound (Pierré et al., 1992).

Co-administration of MDR modulators with the various anti-cancer drugs associated with the MDR phenotype often results in increased net accumulation of drug in MDR cell lines (Luk & Tannock, 1989; Bruno & Slate, 1990; Huet et al., 1993). In the MCF7/DOX cell line, we showed that the cellular content of DOX increased when S9788 and DOX were co-administered over a 1 h period. In addition, the loss of DOX from cells was reduced when S9788 was present during the release phase. Thus, the reversal of resistance after short-term DOX• exposures was the result not only of enhanced cellular accumulation of DOX during DOX exposure, but also of enhanced retention after transfer of cells to DOX-free medium. These results confirmed that the maximum efficacy of S9788 can be achieved by administration of S9788 both during and after administration of the cytotoxic agent. Similar results have been found for VRP (Cass et al., 1989). Moreover, the levels of intracellular DOX have been shown to correlate with cell survival (Keizer et al., 1989; Luk & Tannock, 1989), and our results are in agreement with these data since a relationship was noted between cell exposure to DOX and its cytotoxic effect; thus, the reversal of resistance itself appears to depend directly on the level of accumulation of DOX (Ganapathi et al., 1984; Schuurhuis et al., 1989). Other authors have shown (Huet et al., 1993) that DOX accumulation alone may not predict drug sensitivity; it may be necessary to consider subcellular drug distribution. Since the DOX accumulation measured by HPLC or by flow cytometry is a global detection, it is difficult to identify the various intracellular compartments containing DOX or, more precisely, the DOX molecules involved in the cytotoxicity. Different reports studying the intracellular distribution of DOX have shown that in parental cells fluorescence is predominantly nuclear, whereas in resistant cells fluorescence is distributed in the cytoplasm in distinct punctate regions (Keizer et al., 1989; Schuurhuis et al., 1989; Gervasoni et al., 1991). Other authors have reported that MDR cells exhibit alterations in membrane traffic processes, suggesting endosomal drug trapping (Sehested et al., 1987): DOX could be trapped in vesicles in MDR cells, a phenomenon that would not occur in sensitive cells. It has been shown, using laser scanning confocal fluorescence imaging microscopy, that the presence of a resistance modulator such as VRP is able to increase the intensity of fluorescence of DOX in MDR lines, particularly in the nucleus (Barrand *et al.*, 1993; Coley *et al.*, 1993). Such a technique might be applied to S9788 to confirm the hypothesis expressed by Huet *et al.* (1993) that this drug could be able to decrease the intracellular $IC_{50}$ independently of its efficiency in restoring DOX accumulation, thus reversing intracellular drug tolerance. Such a drug might be able to segregate DOX in subcellular compartments from which it could not reach its nuclear targets.

In conclusion, we have compared the efficacy of various schedules of administration of S9788 in combination with DOX in an MCF7/DOX cell line. The optimum resistance reversion was obtained when cells were incubated with S9788 before, during and after DOX exposure. However, to be compatible with clinical situations, a post-incubation of at least 6 h with the modulator is recommended. The modulation efficiency of this MDR reversal agent was correlated with the DOX cellular content versus time.

### References

AKIYAMA, S., SHIRAISHI, N., KURATOMI, Y., NAKAGAWA, M. & KUWANO, M. (1986). Circumvention of multiple-drug resistance in human cancer cells by thioridazine, trifluoperazine and chlorpromazine. *J. Natl Cancer Inst.*, **76**, 839–844.

BARRAND, M.A., RHODES, T., CENTER, M.S. & TWENTYMAN, P.R. (1993). Chemosensitisation and drug accumulation effects of cyclosporin A, PSC-833 and verapamil in human MDR large cell lung cancer cells expressing a 190k membrane protein distinct from P-glycoprotein. *Eur. J. Cancer*, **29**, 408–415.

BATIST, G., TULPULE, A., SINHA, B.K., KATKI, A.G., MYERS, C.E. & COWAN, K.H. (1986). Overexpression of a novel anionic glutathione transferase in multidrug-resistant human breast cancer cells. *J. Biol. Chem.*, **261**, 15544–15549.

BRUNO, N.A. & SLATE, D.L. (1990). Effect of exposure to calcium entry blockers on doxorubicin accumulation and cytotoxicity in multidrug-resistant cells. *J. Natl Cancer Inst.*, **82**, 419–424.

CAIRO, M.S., SIEGEL, S., ANAS, N. & SENDER, L. (1989). Clinical trial of continuous infusion verapamil, bolus vinblastine, and continuous infusion VP-16 in drug resistant pediatric tumours. *Cancer Res.*, **49**, 1063–1066.

CARL, J. (1989). Drug resistance patterns assessed from tumor marker analysis. *J. Natl Cancer Inst.*, **81**, 1631–1639.

CASS, C.E., JANOWSKA-WIECZOREK, A., LYNCH, M.A., SHEININ, H., HINDENBURG, A.A. & BECK, W.T. (1989). Effect of duration of exposure to verapamil on vincristine activity against multidrug-resistant human leukemic cell lines. *Cancer Res.*, **49**, 5798–5804.

COLDMAN, A.J. & GOLDIE, J.H. (1985). Role of mathematical modeling in protocol formulation in cancer chemotherapy. *Cancer Treat. Rep.*, **69**, 1041–1048.

COLEY, H.M., AMOS, W.B., TWENTYMAN, P.R. & WORKMAN, P. (1993). Examination by laser scanning confocal fluorescence imaging microscopy of the subcellular localisation of anthracyclines in parent and multidrug resistant cell lines. *Br. J. Cancer*, **67**, 1316–1323.

CROS, S., GUILBAUD, N., BERLION, M., DUNN, T.A., REGNIER, G., DHAINAUT, A., ATASSI, G. & BIZZARI, J.P. (1992). *In vivo* evidence of complete circumvention of vincristine resistance by a new triazinoaminopiperidine derivative S9788 in P388/VCR leukemia model. *Cancer Chemother. Pharmacol.*, **30**, 491–494.

DE GREGORIO, M.W., FORD, J.M., BENZ, C.C. & WIEBE, V.J. (1989). Toremifene: pharmacologic and pharmacokinetic basis of reversing multidrug resistance. *J. Clin. Oncol.*, **7**, 1359–1364.

DHAINAUT, A., REGNIER, G., ATASSI, G., PIERRE, A., LEONCE, S., KRAUS-BERTHIER, L. & PROST, J.F. (1992). New triazine derivatives as potent modulators of multidrug resistance. *J. Med. Chem.*, **35**, 2481–2496.

GANAPATHI, R. & GRABOWSKI, D. (1983). Enhancement of sensitivity to adriamycin in resistant P388 leukemia cells by the calmodulin inhibitor trifluoperazine. *Cancer Res.*, **43**, 3696–3699.

GANAPATHI, R., GRABOWSKI, D., ROUSE, W. & RIEGLER, F. (1984). Differential effect of the calmodulin inhibitor trifluoperazine on cellular accumulation retention, and cytotoxicity of anthracyclins in doxorubicin (adriamycin)-resistant P388 mouse leukemia cells. *Cancer Res.*, **44**, 5056–5061.

GERVASONI, J.E., FIELDS, S.Z., KRISHNA, S., BAKER, M.A., ROSADO, M., THURAISAMY, K., HINDENBURG, A.A. & TAUB, R.N. (1991). Subcellular distribution of daunorubicin in P-glycoprotein-positive and -negative drug resistant cell lines using laser-assisted confocal microscopy. *Cancer Res.*, **51**, 4955–4963.

GOTTESMAN, M.M. & PASTAN, I. (1988). Resistance to multiple chemotherapeutic agents in human cancer cells. *Trends Pharmacol. Sci.*, **9**, 54–58.

HUET, S., CHAPEY, C. & ROBERT, J. (1993). Reversal of multidrug resistance by a new lipophilic cationic molecule, S9788. Comparison with 11 other MDR-modulating agents in a model of doxorubicin-resistant rat glioblastoma cells. *Eur. J. Cancer*, **29**, 1377–1383.

KEIZER, H.G., SCHUURHUIS, G.J., BROXTERMAN, H.J., LANKELMA, J., SCHOONEN, W.G.E.J., VAN RIJN, J., PINEDO, H.M. & JOENJE, H. (1989). Correlation of multidrug resistance with decreased drug accumulation, altered subcellular drug distribution, and increased P-glycoprotein expression in cultured SW-1573 human lung cancer cells. *Cancer Res.*, **49**, 2988–2993.

KHAYAT, D., WEIL, M., BENHAMMOUDA, A., VILLEMIN, E., BASTIAN, G., ANTOINE, E., RIXE, O., AUCLERC, G., LUCAS, C., SARKANY, M. & BIZZARI, J.P. (1993). Phase I clinical trial of a new multidrug resistance modulating agent S9788 in combination with vincristine. *Proc. Am. Assoc. Cancer Res.*, **34**, 230.

LEONCE, S., PIERRE, A., ANSTETT, M., PEREZ, V., GENTON, A., BIZZARI, J.P. & ATASSI, G. (1992). Effects of a new triazinoaminopiperidine derivative on adriamycin accumulation and retention in cells displaying P-glycoprotein mediated multidrug resistance. *Biochem. Pharmacol.*, **44**, 1707–1715.

LUCAS, C., BASTIAN, G., FOURNIER, C., SOUDON, J., CLAVEL, M., KHAYAT, D., BENHAMMOUDA, A., SARKANY, M. & BIZZARI, J.P. (1993). Pharmacokinetic support of phase I trials using the reversing agent of multidrug resistance: S9788. *Proc. Am. Assoc. Cancer Res.*, **34**, 391.

LUK, C.K. & TANNOCK, I.F. (1989). Flow cytometric analysis of doxorubicin accumulation in cells from human and rodent cell lines. *J. Natl Cancer Inst.*, **81**, 55–59.

MULLER, C., CHATELUT, E., GUALANO, V., DE FORNI, M., HUGUET, F., ATTAL, M., CANAL, P. & LAURENT, G. (1993). Cellular pharmacokinetics of doxorubicin in patients with chronic lymphocytic leukemia: comparison of bolus administration and continuous infusion. *Cancer Chemother. Pharmacol.*, **32**, 379–384.

OZOLS, R.F., CUNNION, R.E., KLECKER, R.W., HAMILTON, T.C., OSTCHEGA, Y., PARILLO, J.E. & YOUNG, R.C. (1987). Verapamil and adriamycin in the treatment of drug-resistant ovarian cancer patients. *J. Clin. Oncol.*, **5**, 641–647.

PENNOCK, G.D., DALTON, W.S., ROESKE, W.R., APPLETON, C.P., MOSLEY, K., PLEZIA, P., MILLER, T.P. & SALMON, S.E. (1991). Systemic toxic effects associated with high-dose verapamil infusion and chemotherapy administration. *J. Natl Cancer Inst.*, **83**, 105–110.

PEREZ, V., PIERRE, A., LEONCE, S., ANSTETT, M., PROST, J.F. & ATASSI, G. (1993). Caractérisation *in vitro* de l'activité du S9788, un nouveau modulateur de la résistance multidrogue. *Bull. Cancer*, **80**, 310–325.

PIERRE, A., DUNN, T.A., KRAUS-BERTHIER, L., LEONCE, S., SAINT-DIZIER, D., REGNIER, G., DHAINAUT, A., BERLION, M., BIZZARI, J.P. & ATASSI, G. (1992). *In vitro* and *in vivo* circumvention of multidrug resistance by Servier S9788, a novel triazinoaminopiperidine derivative. *Invest. New Drugs*, **10**, 137–148.

RAMU, A., SPANIER, R., RAHAMIMOFF, H. & FUKS, Z. (1984). Restoration of doxorubicin responsiveness in doxorubicin-resistant P388 murine leukaemia cells. *Br. J. Cancer*, **50**, 501–507.

SCHUURHUIS, G.J., BROXTERMAN, H.J., CERVANTES, A., VAN HEIJNINGEN, T.H.M., DE LANGE, J.H.M., BAAK, J.P.A., PINEDO, H.M. & LANKELMA, J. (1989). Quantitative determination of factors contributing to doxorubicin resistance in multidrug-resistant cells. *J. Natl Cancer Inst.*, **81**, 1887–1892.

SEHESTED, M., SKOVSGAARD, T., VAN DEURS, B. & WINTHER-NIELSEN, H. (1987). Increased plasma membrane traffic in daunorubicin-resistant P388 leukaemic cells. Effects of daunorubicin and verapamil. *Br. J. Cancer*, **56**, 747–751.

SOLARY, E., CAILLOT, D., CHAUFFERT, B., CASASNOVAS, R.O., DUMAS, M., MAYNADIE, M. & GUY, H. (1992). Feasibility of using quinine, a potential multidrug resistance-reversing agent, in combination with mitoxantrone and cytarabine for the treatment of acute leukemia. *J. Clin. Oncol.*, **10**, 1730–1736.

TOFFOLI, G., TUMIOTTO, L., GIGANTE, M., DALL'ARCHE, M.G., PENIN, T. & BOIOCCHI, M. (1993). Increased chemosensitivity to doxorubicin of intrinsically multidrug-resistant colon carcinoma cells by prolonged exposure to verapamil. *Eur. J. Cancer*, **29A**, 1776–1778.

TSURUO, T., IIDA, H., TSUKAGOSHI, S. & SAKURAI, Y. (1981). Overcoming of vincristine-resistance in P388 leukemia *in vivo* and *in vitro* through enhanced cytotoxicity of vincristine and vinblastine by verapamil. *Cancer Res.*, **41**, 1967–1972.

TSURUO, T., IIDA, H., TSUKAGOSHI, S. & SAKURAI, Y. (1982). Increased accumulation of vincristine and adriamycin in drug-resistance P388 tumor cells following incubation with calcium antagonists and calmodulin inhibitors. *Cancer Res.*, **42**, 4730–4733.

TSURUO, T., IIDA, H., NOJIRI, M., TSUKAGOSHI, S. & SAKURAI, Y. (1983). Circumvention of vincristine and adriamycin resistance *in vitro* and *in vivo* by calcium influx blockers. *Cancer Res.*, **43**, 2905–2910.

TSURUO, T., IIDA, H., KITATANI, Y., YOKOTA, K., TSUKAGOSHI, S. & SAKURAI, Y. (1984). Effects of quinidine and related compounds on cytotoxicity and cellular accumulation of vincristine and adriamycin in drug-resistant tumor cells. *Cancer Res.*, **44**, 4303–4307.

TWENTYMAN, P.R. (1988). Modification of cytotoxic drug resistance by non-immunosuppressive cyclosporins. *Br. J. Cancer*, **57**, 254–258.

YAHANDA, A.M., ALDER, K.M., FISHER, G.A., BROPHY, N.A., HALSEY, J., HARDY, R.I., GOSLAND, M.P., LUM, B.L. & SIKIC, B.I. (1992). Phase I trial of etoposide with cyclosporine as a modulator of multidrug resistance. *J. Clin. Oncol.*, **10**, 1624–1634.